# Orthokeratology and Low-Intensity Laser Therapy for Slowing the Progression of Myopia in Children

**DOI:** 10.1155/2021/8915867

**Published:** 2021-01-27

**Authors:** Fen Xiong, Tian Mao, Hongfei Liao, Xiaoqin Hu, Lei Shang, Li Yu, Nana Lin, Liang Huang, Yunmin Yi, Rui Zhou, Xueyun Zhou, Jinglin Yi

**Affiliations:** Affiliated Eye Hospital, Nanchang University, Nanchang 330006, China

## Abstract

Orthokeratology (OK) is widely used to slow the progression of myopia. Low-level laser therapy (LLLT) provides sufficient low energy to change the cellular function. This research is aimed at verifying the hypothesis that LLLT treatment could control myopia progression and comparing the abilities of OK lenses and LLLT to control the refractive error of myopia. Eighty-one children (81 eyes) who wore OK lenses, 74 children (74 eyes) who underwent LLLT treatment, and 74 children (74 eyes) who wore single-vision distance spectacles for 6 months were included. Changes in axial length (AL) were 0.23 ± 0.06 mm for children wearing spectacles, 0.06 ± 0.15 mm for children wearing OK lens, and −0.06 ± 0.15 mm for children treated with LLLT for 6 months. Changes in subfoveal choroidal thickness (SFChT) observed at the 6-month examination were −16.84 ± 7.85 *μ*m, 14.98 ± 22.50 *μ*m, and 35.30 ± 31.75 *μ*m for the control group, OK group, and LLLT group, respectively. Increases in AL at 1 month and 6 months were significantly associated with age at LLLT treatment. Changes in AL were significantly correlated with the baseline spherical equivalent refraction (SER) and baseline AL in the OK and LLLT groups. Increases in SFChT at 1 month and 6 months were positively associated with age at enrolment for children wearing OK lens. At 6 months, axial elongation had decelerated in OK lens-wearers and LLLT-treated children. Slightly better myopia control was observed with LLLT treatment than with overnight OK lens-wearing. Evaluations of age, SER, and AL can enhance screening for high-risk myopia, improve the myopia prognosis, and help determine suitable control methods yielding the most benefits.

## 1. Introduction

Myopia is a global epidemic, which is especially prevalent in East Asia [1]. Myopia, especially in higher levels, results in an increased risk of complications such as retinal detachment, myopic macular degeneration, glaucoma, cataracts, and permanent vision loss [2]. Therefore, a safe, reliable, and effective therapy to slow the progression of myopia would be advantageous for millions of individuals.

Oxidative stress, inflammation, and apoptosis may be key factors in the myopic regulatory pathways [3–6]. The low levels of 5-MTHF in myopia patients may lead to the increase of homocysteine, which closely correlated with oxidative stress, inflammation, and cellular apoptosis [5]. Furthermore, exogenous bFGF effectively ameliorates the excessive axial elongation in chronic form-deprivation myopia in chicks by suppressing retinal neuron apoptosis [3]. Finding a method to suppress the cell apoptosis may be an effective way to control the axial elongation. Thus, we first focused on low-level laser therapy (LLLT) as a new method that contributes to restricting the progression of myopia by preventing cell apoptosis, thereby minimizing inflammation and increasing cell turnover [7, 8].

Recent studies suggest that normal ocular growth and refractive development could be influenced by the spectral composition of ambient light in a variety of ways through chromatic cues [9, 10]. The laser is a device that is widely used for biomedical applications; it creates a pure, intense, monochromatic, and coherent collimated light beam [11, 12]. Laser therapy results in a broad range of molecular, cellular, and tissue effects [13]. LLLT differs from high-power laser therapy because it uses low levels of red and near-infrared light. Its wavelength ranges from 600 nm to 1100 nm, and its output can reach 500 mW [7]. Therefore, it is defined as a type of phototherapy that produces sufficiently low energy to induce a stimulus response in tissues without changing the temperature of the surrounding tissues [7].

Various studies have demonstrated that modern orthokeratology (OK) is a key intervention for myopia control that has great effectiveness in children; however, the mechanism remains unclear [14–16]. The OK lens is characterized by its reverse-geometry design, which can form peripheral defocus in the central and peripheral retina by remodeling the cornea and changing the image quality [17, 18]. Recent studies in myopic chicks have demonstrated that the choroid tends to thin with myopia development before alterations in scleral growth [19, 20]. Therefore, the role of the choroid has been emphasized in the OK lens control mechanism [21, 22].

This study describes the research design and clinical 6-month follow-up examinations of children who wore OK lens, underwent LLLT treatment, or wore single-vision distance spectacles. This study is aimed at verifying the hypothesis that LLLT treatment could control myopia progression and comparing the abilities of OK lenses and LLLT to control the progression of myopia.

## 2. Participants and Methods

### 2.1. Participants

**Figure 1:**
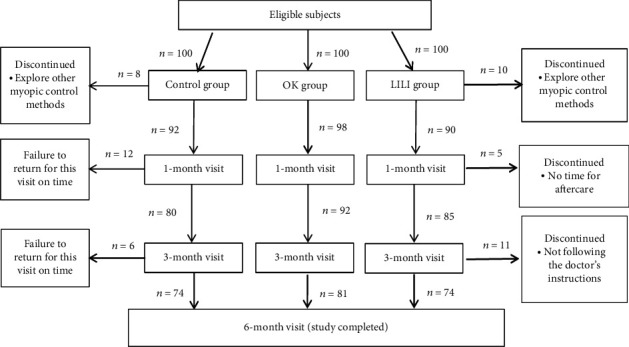
Flow chart of participant assignment.

All participants had myopia; their data were collected from the Outpatient Clinic of the Nanchang University Affiliated Eye Hospital, Nanchang, China, from September 2018 to April 2019. The inclusion criteria were age 6 to 16 years, spherical power plus half cylinder power, spherical equivalent refractive error (SER) ≤ −0.50 D after cyclopentanone use, and 10 to 21 mmHg noncontact tonometer intraocular pressure. The exclusion criteria were presence of ocular or systemic diseases such as strabismus, amblyopia, and cardiac respiratory illness. Children already using OK and/or other myopia control modalities, except for wearing spectacles, were also excluded. We recruited a total of 300 children that were randomly assigned to control (*n* = 100), OK (*n* = 100), or LLLT (*n* = 100) groups. Consequently, 229 children completed the study (Figure 1). A total of 74 children 7 to 14 years of age (mean age, 10.33 ± 2.03 years; 54% male) were included in the control group. A total of 81 children 8 to 14 years of age (mean age, 10.88 ± 1.92 years; 54% male) were included in the OK group. A total of 74 children 7 to 15 years of age (mean age, 10.22 ± 2.38 years; 54% male) were included in the LLLT group. Differences in sex, age, SER, axial length (AL), and subfoveal choroidal thickness (SFChT) at baseline were not significant among the three groups.  Table 1 lists the general characteristics of the enrolled children.

The present study was performed in compliance with the principles of the *Declaration of Helsinki* and was approved by the ethical committee of Nanchang University Clinical Research Centre (2018-KY-03). Parents understood the benefits and risks of this study before providing signed informed consent on behalf of their children.

### 2.2. Study Procedures

Researchers performed detailed ophthalmological examinations before treatment (baseline) and at every subsequent appointment; these examinations included uncorrected and corrected visual acuity tested at a distance of 4 m using a retro-illuminated Early Treatment of Diabetic Retinopathy Study chart, cycloplegic refraction testing (1% cyclopentolate hydrochloride), the spherical equivalent (SE) determined (obtained with the following formula: SE = spherical + astigmatism/2), slit-lamp examination (slit lamp; Haag-Streit, Köniz, Switzerland), ocular movement testing, tonometry (model NT-4000; Nidek Inc., Fremont, CA, USA), fundoscopy, AL measurements (Carl Zeiss Meditec Inc., Dublin, CA, USA), corneal endothelial cell density testing, and optical coherence tomography (OCT) (Carl Zeiss Meditec Inc., Dublin, CA, USA).

To avoid the effects of circadian rhythm on the results, OCT scanning was performed twice by the same investigator between 8:00 am and 2:00 pm at baseline, 1-month, 3-month, and 6-month follow-ups. Two independent skilled professionals measured the SFChT using a linear measurement program during the OCT scan. To increase the visibility of the choroid, the enhanced depth imaging mode was used. We defined the thinnest part of the macula in the image as the fovea. The SFChT was measured from the outermost part of the retinal pigment epithelium to the inner layer of the choroidoscleral interface.

Children in the control group wore single-vision distance spectacles the entire day and returned for detailed ophthalmological examinations after 1, 3, and 6 months. Children in the OK group were fitted with OK lenses by our fitting staff. The OK lenses (Euclid Systems Ortho-k; Euclid System Corp., Herndon, VA, USA) used in the present study were made of rigid gas-permeable material (Boston Equalens II; Bausch + Lomb, Laval, Quebec, Canada). The diameter of the lenses ranged from 10.2 to 11 mm. The lens consisted of a central base curve with a 6.2 mm optic zone diameter, 0.5 mm wide reverse curve, 1.2 mm wide alignment curve, and 0.5 mm wide peripheral curve. They wore them every night for at least 7 consecutive hours to guarantee myopia control. Children returned for ophthalmological examinations after 1, 3, and 6 months. In addition to the aforementioned examinations, we also used a corneal fluorescein stain to determine any complications and check the corneal topography to ensure the correct fit of the OK lens.

Children in the LLLT group wore single-vision distance spectacles the entire day and underwent LLLT (power, 2 ± 0.5 mW; wavelength, 650 nm; Ya Kun Optoelectronic Co., Ltd., Wuhan, China) twice per day for 3 minutes each session, with at least a 4-hour interval between sessions. There were no specific guidelines for room illumination. After the first measurement session, each child returned for follow-up examinations at 1, 3, and 6 months and completed all the aforementioned examinations.

### 2.3. Data Analysis

Statistical analyses were performed using IBM SPSS statistics version 23.0 (IBM Co., Armonk, NY, USA). Only the data of the left eyes were used. All values were first tested for normality and are presented as the mean ± the standard deviation unless otherwise stated.

One-way analysis of variance (ANOVA) followed by Tukey's *post hoc* tests were used to analyze the differences in basic variable data of the subgroups. A comparison of sexes among the three groups was performed using the chi-square test. Changes in SER, AL, and SFChT between baseline and each follow-up visit were analyzed by repeated-measures ANOVA. The Greenhouse-Geis test was used if the sphericity assumption was violated. The main effects of time, group, and the interaction of effect time and group were included in the model. Correlations between changes in parameters at 6 months and baseline factors were analyzed using the Pearson correlation analysis. To study the association of AL/SFChT changes at 6 months with baseline factors in all groups, we applied stepwise multiple linear regression models. *p* value < 0.05 was defined as statistically significant.

## 3. Results

**Figure 2:**
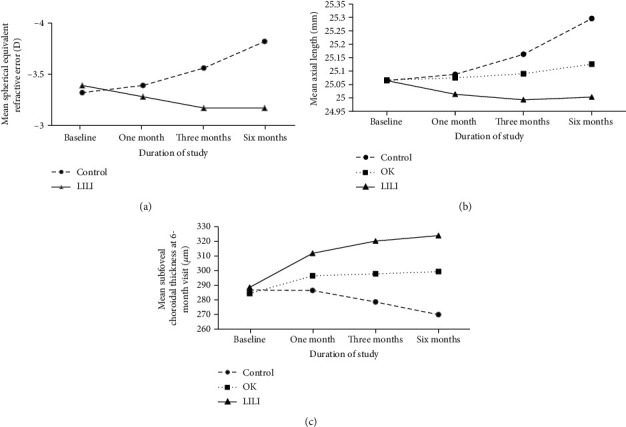
Time courses of mean spherical equivalent refractive error (SER), axial length (AL), and subfoveal choroidal thickness (SFChT). Error bars represent the standard deviation. (a) Time courses of mean SER in control group, and LLLT group. (b) Time courses of mean AL in control group, OK group, and LLLT group. (c) Time courses of mean SFChT in control group, OK group, and LLLT group.

The mean SER decreased slightly over time from baseline to the 6-month follow-up in the LLLT group, but it increased from baseline to the 6-month follow-up in the control group. This disparity between the control and LLLT groups was statistically significant. The mean AL increased in the control group and OK group, but decreased slightly in the LLLT group. These changes differed significantly from each other over time. During the same period, the mean AL was shorter in the LLLT group than that in the other two groups. The SFChT in the LLLT and OK groups compared to that in the control group was thicker at each examination, and the difference was statistically significant (Table 2, Figure 2).

Table 3 displays the different timetables of changes in parameters at each sampling point for the three groups. Changes in SER were significantly different in the control group and LLLT group at the time of the study (*p* < 0.001). At the 1-month follow-up, the mean changes in SER were −0.07 ± 0.11, −0.24 ± 0.16, and −0.50 ± 0.24 D in the control group and 0.11 ± 0.17, 0.22 ± 0.32, and 0.21 ± 0.34 D in the OK group at 1 month, 3 months, and 6 months, respectively. Increases in AL were significantly smaller in children wearing OK lens than in the control group at the 3-month follow-up and 6-month follow-up, but changes at the 1-month follow-up were not (*p* = 0.184). Decreases in AL in the LLLT group differed significantly from those in the control and LLLT groups at each sampling point. Increases in SFChT at 1 month, 3 months, and 6 months were significantly smaller in OK lens-wearers than in the LLLT group, whereas the SFChT in the control group significantly decreased.

**Figure 3:**
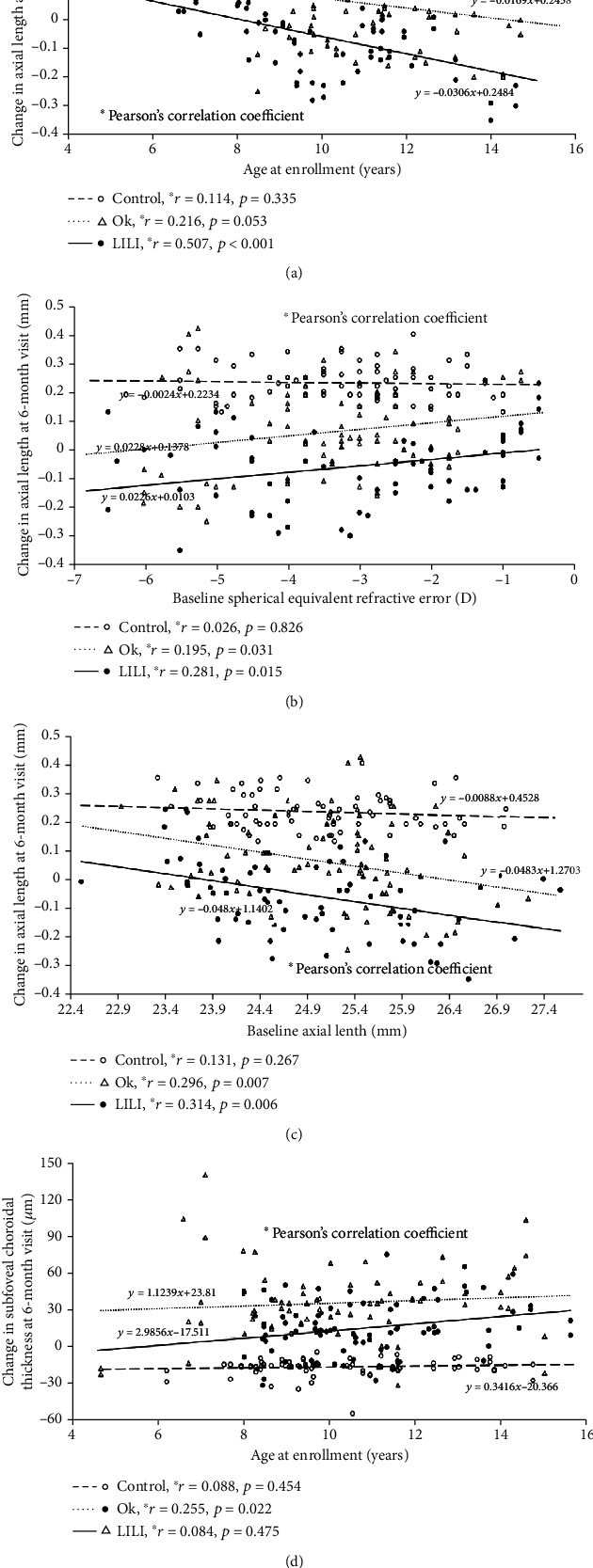
Scatter plots of the change in subfoveal choroidal thickness (SFChT) and axial length (AL) versus the baseline factors in the control group, OK group, and LILI group at the 6-month visit. (a) Scatter plots of the change in AL and age at enrolment at the 6-month visit. (b) Scatter plots of the change in AL and the baseline spherical equivalent refractive error at the 6-month visit. (c) Scatter plots of the change in AL and the baseline AL at the 6-month visit. (d) Scatter plots of the increase in the SFChT and the age at enrolment at the 6-month visit.

To understand the relationship between parameter changes and baseline factors, Pearson's correlation coefficient was used. The scatter plot graph of the increase in AL at 6 months and age of the groups is shown in Figure 3(a). There were no statistically significant correlations in the control group (*r* = −0.114; *p* = 0.335) and the OK group (*r* = −0.216; *p* = 0.053) in terms of increased AL at 6 months and age at enrolment. However, in the LLLT group, there was a significant correlation between these two parameters at 1 month and 6 months (1 month: *r* = −0.307 and *p* = 0.008; 6 months: *r* = −0.507 and *p* < 0.001). We also found a significant correlation between the change in AL and baseline SER in the OK and LLLT groups (OK group: *r* = 0.195 and *p* = 0.031; LLLT group: *r* = 0.281 and *p* = 0.015, Figure 3(b)). A significant correlation was also found between the changes in AL and baseline AL in the OK lens group (*r* = −0.296 and *p* = 0.007) and the LLLT group (*r* = −0.314; *p* = 0.006, Figure 3(c)).  Figure 3(d) presents the scatter plot graph of the increase in SFChT over 6 months and age in these groups. The increase in SFChT had a strong positive relationship with age of enrolment for OK lens-wearers not only at the 1-month follow-up but also at the 6-month follow-up (1 month: *r* = 0.343 and *p* = 0.002; 6 months: *r* = 0.255 and *p* = 0.022); the increase in SFChT was larger in individuals who were older.

Changes in AL over 6 months had a strong positive connection with baseline AL according to the multiple linear regression analysis, and a significant association was modified by the sex effect in the multivariate model. The formula used to determine the changes in AL over 6 months was as follows: 0.007∗baseline AL mm + 0.034∗sex (male = 1, female = 2; *R*^2^ = 0.936, *p* < 0.01). A negative correlation between baseline AL and changes in AL over 6 months according to the multiple linear regression was found in the OK group. In this model, baseline AL explained 15.4% of the variance (*β* = −0.059; *p* < 0.01). Among the children in the LLLT group, the baseline AL and age were independently related to changes in AL over 6 months (*R*^2^ = 0.387). According to the model, shorter AL and older age were closely linked to fewer increases in AL (baseline AL: *β* = 0.013; age: *β* = −0.03; all *p* < 0.01).

Independent factors associated with changes in SFChT over 6 months were explored using a multiple regression analysis. In the model, there was a relationship between baseline AL and changes in SFChT over 6 months for the control group and LLLT group, but not for the OK group (control group: *β* = −0.67, *R*^2^ = 0.819, *p* < 0.01; LLLT group: *β* = 1.408, *R*^2^ = 0.557, *p* < 0.01). The significant correlation between age and changes in SFChT over 6 months in the OK group was confirmed by a multiple linear regression analysis (*β* = 1.424; *R*^2^ = 0.342; *p* < 0.01). However, these two parameters were not relevant to the other two groups.

## 4. Discussion

To our knowledge, this is the first study specifically designed to test the hypothesis that LLLT can control the progression of myopia in children and to compare the effects of wearing OK lenses and undergoing LLLT to control myopia progression in children. Our results showed that LLLT can help control AL elongation and slow myopia progression better than OK lens-wearing (Table 2, Figure 2).

Most studies exploring OK lens-wearing have considered changes in AL as representative of myopia. The AL was defined as the distance from the corneal vertex to the retinal pigment epithelium. According to our results, wearing OK lenses is a more effective method of preventing axial elongation over the course of 6 months when compared to wearing single-vision glasses. As reported previously, AL in children after 1, 3, and 6 months of wearing OK lenses increases by 0.02 mm [23, 24], 0.02 mm [22], and 0.02-0.12 mm [23–25], respectively, which is consistent with our results (Table 3). At the time of our 6-month follow-up, the results of the single-vision spectacle lens group indicated an increase in AL of 0.23 ± 0.06 mm, which is consistent with previous studies (0.18-0.24 mm) [23–25], and the LLLT group exhibited a decrease in AL of −0.06 ± 0.15 mm. However, OK lens-wearing children had an AL increase of 0.06 ± 0.15 mm; therefore, LLLT treatment more effectively slowed the progression of myopia than OK lens treatment. Three principles formed the basis of the therapeutic LLLT treatments: (1) minimizing inflammation and edema and improving tissue microcirculation without puncturing the skin or entering a body cavity, (2) promoting neurological damage, and (3) treating neurological disorders [26]. Currently, vast quantities of empirical evidence have indicated that oxidative stress and inflammation may account for the altered regulatory pathways in myopia and that oxidative damage associated with hypoxic myopia can alter the neuromodulation of nitric oxide and dopamine during eye growth [4, 5]. Analyzing the possible mechanisms of the inhibitory effects of LLLT treatment on myopia could help to protect patients from the effects of oxidative stress and decrease inflammation that accompanies myopia [27]. LLLT has maximal effects on the nitric oxide system and decreases the severity of oxidative stress in both animal studies [28] and clinical studies [29–31]. LLLT may reduce the levels of inflammatory cytokines such as interleukin- (IL-)1 and tumor necrosis factor-*α* by inhibiting them [32]. Furthermore, severe myopia could significantly increase the levels of IL-1 and IL-6 [33, 34], which could be associated with the myopic control mechanism.

The choroid has a variety of functions, including nourishing the retina [35] and changing the refractive state through the modulation of its thickness [18, 36]. Furthermore, the choroid has a crucial role in relaying signals derived from the retina to the sclera, further altering the synthesis of scleral extracellular matrix and changing the ocular size, resulting in refractive changes that have a vital function in the aetiology of myopia [19, 20]. Enhanced depth imaging OCT is a novel noninvasive imaging tool that produces high-resolution real-time images that allow visualization of the choroid *in vivo*, thereby allowing for a better understanding of changes in the choroid [37, 38]. Several studies have confirmed that visual signals not only change the process of emmetropization but also change the choroidal thickness in primates [39]. Furthermore, the most credible mechanism by which the OK lens could reduce myopia progression appears to be the increased myopic defocus in the central and peripheral retina [40, 41], but the exact mechanism remains unclear. Some researchers have speculated that choroidal thickening may contribute to the altered retinal defocus profile, but have reported conflicting results [22, 23, 42, 43]. Therefore, we chose to investigate SFChT using enhanced depth imaging OCT as another ocular biometric parameter to appraise the effects of control on slowing the progression of myopia.

In our study, OK and LLLT treatment increased the SFChT over time, and the rate was high at the 1-month examination (12.14 ± 15.30 *μ*m and 23.23 ± 24.70 *μ*m, respectively); then, it slowed compared with the increase resulting from spectacle wear (−0.36 ± 2.09 *μ*m). Similar results were reported in individuals who wore OK lenses for 3 weeks [42] or 6 months [24], although no choroidal changes were found in another study [21]. These studies showed greater thickening of the choroid in patients wearing OK lenses than in spectacle wearers (approximately 16-21.8 *μ*m) [23, 42]; however, this effect peaked after 1 month of treatment, and the amplitude of variation in choroidal thickening remained unchanged at the 6-month and 12-month examinations [23].

Changes in the SFChT at 1 month had a strong positive correlation with the age at which OK lens-wearing was started, which meant that older children showed thicker change in SFChT, and the positive effects persisted until the 6-month follow-up. Changes in AL were negatively correlated with baseline age only at the 6-month follow-up, with borderline significance (*p* = 0.053). Older children showed increased SFChT changes and slower axial elongation compared with younger children wearing OK lens, which was consistent with some randomized trials of OK treatment to reduce myopia progression [44, 45]. Changes in AL after 1 month of LLLT treatment were also significantly correlated with baseline age, and the negative effects persisted until 6 months. Although older children showed slower axial elongation than younger children after undergoing LLLT treatment, changes in the SFChT showed no significant correlation with age. Older children showed no advantage in SFChT changes when treated with LLLT. Thus, the mechanism for LLLT controlling axial elongation may not be by directly affecting the choroid, but through another pathway. The role of age in the effects on SFChT has been a divisive issue. Many authors have reported that increasing age is related to decreased SFChT in adults [46, 47]. However, in a population with emmetropia, from early childhood to adolescence, the SFChT increased significantly [48, 49]. Another study reported a positive relationship between SFChT and age for those with emmetropia and hyperopia [50].

Baseline SE and baseline AL might be predictive factors for AL changes in myopic individuals treated with OK lenses or LLLT. However, studies have shown conflicting results regarding the relationship between SER and changes in AL [44, 51, 52]. We found that more myopic diopter and longer AL were significantly related to decreased AL changes after wearing OK lenses and LLLT treatment. These results are in line with several studies that have confirmed that OK lenses provide more advantages for individuals with higher degrees of myopia and longer AL and that lower myopia at the start of OK lens-wearing makes the design less effective than it is for high myopia [52]. The authors hypothesized that this is due to the greater degree of corneal steepening in the midperiphery of eyes with higher myopia and greater peripheral retinal defocus, which slows myopia progression [51, 53]. Higher baseline myopia before LLLT treatment was associated with slower axial elongation compared to the control group. This may be due to the high levels of certain cytokines (IL-1, IL-6) in highly myopic eyes [33, 34], which absorb more energy and thus increase the effects of LLLT.

The most apparent limitation of our study was its short duration. Therefore, a long-term study of outcomes of all 3 groups is warranted to compare the effects and side effects of OK lens-wearing to those of LLLT treatment.

## 5. Conclusions

This study is the first to utilize LLLT to slow the progression of myopia and to compare OK lens-wearing and LLLT treatment with single-vision spectacle lens-wearing. Our study found that OK lens-wearing and treatment with LLLT more effectively slowed the progression of myopia than single-vision distance spectacles after a 6-month period of treatment. We also found some factors that were significantly correlated with changes in AL and SFChT. Therefore, an evaluation of basic characteristics, such as age, SE, and AL, can lead to advanced screening for high-risk myopia, predictions of myopia prognoses, and choosing suitable control methods for myopia that will provide the most benefit for children.

## Figures and Tables

**Table 1:** Baseline characteristics of study groups.

Characteristics	Control (*n* = 74)	OK (*n* = 81)	LILI (*n* = 74)	*p* value
Sex (male : female)	40 : 34	44 : 37	40 : 34	0.412^a^
Age	10.33 ± 2.03	10.88 ± 1.92	10.22 ± 2.38	0.114^b^
SER (D)	−3.32 ± 1.36	−3.42 ± 1.28	−3.39 ± 2.17	0.937^b^
AL (mm)	25.07 ± 0.87	25.07 ± 0.92	25.07 ± 1.15	0.99^b^
SFCHT (*μ*m)	286.81 ± 63.67	284.36 ± 72.58	288.61 ± 59.59	0.921^b^

SER: spherical equivalent refractive error; AL: axial length; SFCHT: subfoveal choroidal thickness. ^a^Chi-square test. ^b^One-way ANOVA.

**Table 2:** Parameters at different sampling points (mean ± SD).

Parameters		Control (*n* = 74)	OK (*n* = 81)	LILI (*n* = 74)
SER (D)	Baseline	−3.32 ± 1.36	−3.42 ± 1.28	−3.39 ± 2.17
	One month	−3.39 ± 1.35		−3.28 ± 2.14
	Three months	−3.56 ± 1.37		−3.17 ± 2.13
	Six months	−3.82 ± 1.37		−3.17 ± 2.14
	*F* main effect/*p* value: 27.82/<0.001			
	*F* crossover effect/*p* value: 128.80/<0.001			

AL (mm)	Baseline	25.07 ± 0.87	25.07 ± 0.92	25.06 ± 1.14
	One month	25.09 ± 0.87	25.07 ± 0.91	25.01 ± 1.14
	Three months	25.16 ± 0.87	25.09 ± 0.88	24.99 ± 1.11
	Six months	25.30 ± 0.86	25.13 ± 0.89	25.00 ± 1.11
	*F* main effect/*p* value: 67.21/<0.001			
	*F* crossover effect/*p* value: 62.86/<0.001			

SFChT (*μ*m)	Baseline	286.81 ± 63.67	284.36 ± 72.58	288.61 ± 59.59
	One month	286.45 ± 63.61	296.49 ± 72.61	311.84 ± 67.08
	Three months	278.59 ± 63.64	297.81 ± 73.62	320.18 ± 66.61
	Six months	269.97 ± 64.11	299.33 ± 73.65	323.91 ± 65.63
	*F* main effect/*p* value: 53.00/<0.001			
	*F* crossover effect/*p* value: 64.42/<0.001			

SER: spherical equivalent refractive error; AL: axial length; SFChT: subfoveal choroidal thickness.

**Table 3:** Change in parameters at each sampling point (mean ± SD).

Parameters		Control (*n* = 74)	OK (n = 81)	LILI (*n* = 74)	*F* value	*p* value
Change in SER (D)	1 month	−0.07 ± 0.11		0.11 ± 0.17	11.24	<0.001^a^
	3 months	−0.24 ± 0.16		0.22 ± 0.32	11.61	<0.001^a^
	6 months	−0.50 ± 0.24		0.21 ± 0.34	6.58	<0.001^a^

Change in AL (mm)	1 month	0.02 ± 0.02	0.01 ± 0.08	−0.05 ± 0.07	26.15	<0.001^b^
	3 months	0.10 ± 0.04	0.02 ± 0.17	−0.07 ± 0.12	35.92	<0.001^b^
	6 months	0.23 ± 0.06	0.06 ± 0.15	−0.06 ± 0.15	98.13	<0.001^b^

Change in SFChT (*μ*m)	1 month	−0.36 ± 2.09	12.14 ± 15.30	23.23 ± 24.70	36.65	<0.001^b^
	3 months	−8.22 ± 3.24	13.46 ± 19.46	31.58 ± 31.72	63.50	<0.001^b^
	6 months	−16.84 ± 7.85	14.98 ± 22.50	35.30 ± 31.75	97.48	<0.001^b^

SER: spherical equivalent refractive error; AL: axial length; SFChT: subfoveal choroidal thickness. ^a^Independent sample *t*-test. ^b^One-way ANOVA.

## Data Availability

The data used to support the findings of this study are available from the corresponding author upon request.
